# Correlation and epidemiologic distribution of emerging coinfections of *Plasmodium falciparum* and dengue virus among febrile children in malaria-endemic zones in western Kenya

**DOI:** 10.1016/j.ijregi.2025.100737

**Published:** 2025-08-22

**Authors:** Jack Ogony, Judith Mangeni, George Ayodo, Diana Menya, Ivy Akinyi, Ben Oyugi, Arthy Yongo, Fordrane Okumu, Charles Lwanga, Fredrick Oluoch, Simon Karanja

**Affiliations:** 1 Department of Environmental Health and Disease Control, Jomo Kenyatta University of Agriculture and Technology, Nairobi, Kenya; 2 Department of Epidemiology and Biomedical Statistics, Moi University, Eldoret, Kenya; 3 Department of Public Health and Community Health, Jaramogi Oginga Odinga University of Science and Technology, Kisumu, Kenya; 4 Adaptive Model for Research and Empowerment in Communities (AMREC), Kisumu, Kenya; 5 Department of Health and Sanitation, Ministry of Health, Kisumu, Kenya

**Keywords:** Correlation, Coinfection, Dengue fever, Febrile illness, *P. falciparum*, Vector-borne

## Abstract

**Objectives:**

To determine correlation and epidemiological distribution of emerging coinfections of *Plasmodium falciparum* and dengue fever among febrile children in malaria endemic zones in western Kenya.

**Methods:**

Prospective cohort study. This study was conducted in Kisumu and Busia Counties in western Kenya. Kisumu County is an urban setting, whereas Busia has peri-urban and rural settings. All the level 2-4 public health facilities in Kisumu central subcounty and Bunyala subcounty in Busia were included. The number of participants recruited per facility was based on the facility level proportions. From each facility level, 18 and 19 participants were recruited in Kisumu and Busia, respectively. A total of 380 febrile children aged below 5 years who screened positive for malaria and/or dengue virus and whose parents/guardians gave consent to participate were recruited. Demographic parameters were simultaneously analyzed. Statistical analysis was performed using the chi-square test.

**Results:**

Of the 1004 participants screened, 380 turned seropositive for either malaria or dengue or coinfected. The overall disease burden was 37.8% (380 of 1004). The prevalence of *P. falciparum*, dengue and coinfections were 21.4% (215 of 1004), 8.9% (90 of 1004), and 7.5% (75 of 1004), respectively. Busia had the highest *P. falciparum–*only infections (31% [118 of 380]), whereas Kisumu had the highest dengue-only infections (16.6% [63 of 380]). The distribution of these diseases was not random across the counties but rather associated with the location (X² = 19.45, *P* <0.001). At the county level, differences in prevalence were statistically significant for dengue (*P* = 0.038) and malaria (*P* <0.001).

**Conclusion:**

This study finding is suggestive of an active spread of dengue virus infections, leading to coinfections in this geographical region where malaria is endemic. The high temperatures, precipitation, and humidity experienced around Lake Victoria is favoring mosquito vector multiplication, hence the sustained mosquito transmitted disease burden. It is also important to educate clinicians on the differential diagnoses for appropriate case management because the disease patterns vary meaningfully between the two study sites.

## Highlights

• *Plasmodium* and dengue virus coinfections have serious and fatal health outcomes.
• Dengue and malaria coinfection are rising mosquitoborne disease and leading acute febrile illness among children.
• Coinfections are an indication of the disease overlap and diagnosis challenges.
• The climatic conditions in the region enhances vector fecundity, threatening epidemics.

## Introduction

**Figure 1:**
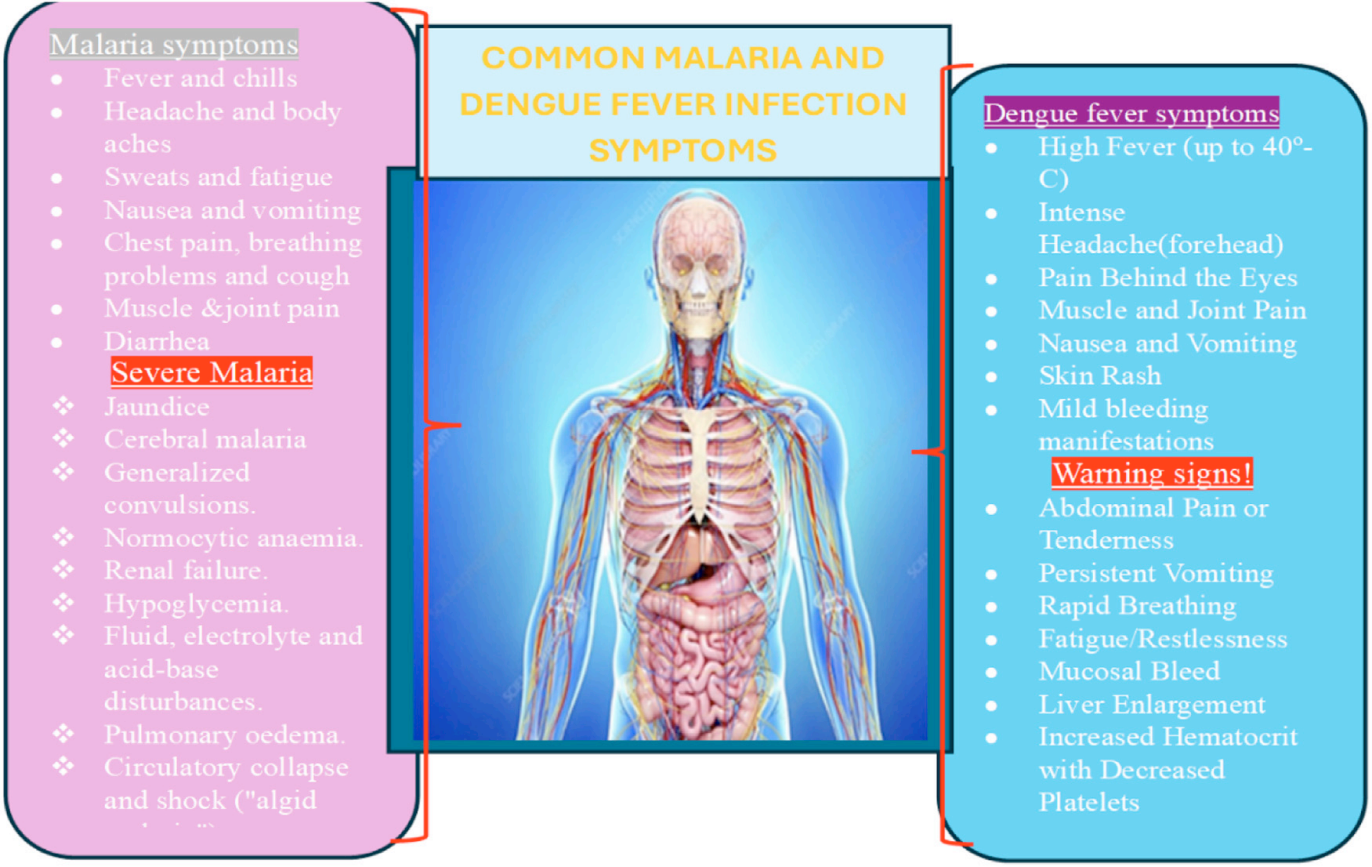
Common malaria and dengue fever signs and symptoms.

Worldwide, dengue and malaria are mosquito-borne illnesses that are a public health threat, especially in tropical and subtropical climates [1]. The spread of these diseases has drawn great concerns because of their recurring outbreaks, with some already endemic in certain areas, causing millions of cases every year [2]. D viral infection and malaria parasitic infection cause acute febrile illness, with quite similar symptoms and signs and may not be clinically distinguishable [3]. (Figure 1). The *Aedes mosquito*, especially the *Aedes aegypti* and *Aedes albopictus* species, are the primary vectors for the dengue virus (DENV) that causes dengue disease [4]. Serotypes DENV-1, DENV-2, DENV-3, and DENV-4 are the four forms of the DENV [5]. Malaria, on the other hand, is an acute febrile illness caused by *Plasmodium* parasites transmitted to humans via the bites of infected female *Anopheles* mosquitoes [6]. There are multiple species of *Plasmodium* that infect people; however, *Plasmodium falciparum* and *Plasmodium vivax* are the most dominant and potentially lethal forms [7]. The increasing burden has been linked with climate change; although Africa is the least greenhouse gas emitter, it is affected severely by the impacts of climate change [8]. Other factors that lead to the increased emergence and re-emergence of infections include urbanization, deforestation, and agricultural settlements in peri-urban areas [9]. Climate change not only expands the geographical range and transmission seasons of malaria but also increases vector’s population and biting rates [10].

The distribution and transmission of mosquito-borne infections often overlap; this commonly results in coinfections [11,12]. Coinfection with *Plasmodium* and DENV infectious species could have serious and fatal outcomes if not promptly diagnosed and treated [3]. According to the 2023 World Malaria Report, the number of global malaria cases was estimated at 263 million, with an incidence of 60.4 cases per 1000 population at risk, an increase of 11 million cases from the previous year, signifying a rise in incidence from 58.6 cases per 1000 in 2022 [13]. Sub-Saharan Africa continues to carry the heaviest burden of the disease, accounting for an estimated 94% of global malaria burden in 2023 [14]. Similarly, according to the World Health Organization, over 7.6 million dengue cases were reported in 2024, including 3.4 million confirmed cases, over 16,000 severe cases, and over 3000 deaths. This disease is found primarily in urban and peri-urban areas in tropical and subtropical climates, putting approximately half of the world’s population at risk, in which between 100 and 400 million cases of the disease occur annually [15].

Although malaria is treatable, any delay in treatment may result in severe disease and even mortality. However, there is no globally rolled vaccine or specific treatment for dengue. In recent years, both diseases have become the leading cause of morbidity and mortality in tropical and subtropical areas and approximately two-fifths of the world’s population who live in these areas that are at risk [15,16]. In Kenya, malaria remains a major public health problem, with 75% of the population being at risk [17]. In 2023, Kenya had an estimated 3.3 million malaria cases and 11,800 deaths; those living in western Kenya have an especially high risk of malaria [18]. Malaria epidemic in this region is influenced by climate change; high altitudes, shift in weather temperatures, and stagnant waters, which provide a breeding ground for mosquitoes. The transmission peaks are known to be between March to May and October to December during the rainy periods [19]. This is currently changing due to climate change, which favors vector multiplication, irrespective of the rainy seasons and distribution beyond the endemic regions [20]. For instance, a study done in 2022 by the National Malaria Control Program and Kenya Medical Research Institute reported an invasion of *Anopheles stephensi* in Marsabit and Turkana, regions historically known as having low or no malaria transmission [21]. Furthermore, there are also evidences of dengue fever in Kenya, with several documented epidemics and outbreaks in different locations; the most recent outbreak reported was from Mombasa in May 2017 [22].

Infections from DENV and *P. falciparum* are exasperating enough individually. However, the contemporaneous presence of both infections in an individual is dire. According to the World Health Organization guidelines, dengue viral and malaria parasitic coinfection in an individual is regarded as a “severe malaria” case [7]. In the rural, peri-urban, and urban areas of western Kenya, both vectors (*Aedes* and *Anopheles*) are present throughout the year [23]; therefore, the existence of malaria–dengue coinfection in an individual cannot be ruled out. There are limited documented data on epidemiologic trends and correlations of malaria and dengue fever coinfections; likewise, there are limited data on dengue fever alone or coinfections in malaria-endemic regions. Therefore, this study aimed to determine the correlation and epidemiologic distribution of emerging malaria and DENV coinfections among febrile children in the western part of Kenya.

## Materials and methods

### Study site

Kisumu County (site A) is a port city in western Kenya, located on a bay on the eastern shore of Lake Victoria at an altitude of 1131 m. It is the headquarters of Kisumu County. Kisumu County is one of the 47 counties in Kenya, lying within longitudes 33° 20’E and 35° 20’E and latitudes 0°20’south and 0°50’south. It is bordered by Homa Bay County to the south, Nandi County to the northeast, Kericho County to the east, Vihiga County to the northwest, and Siaya County to the west. According to the 2019 National Census, Kisumu County has a population of 1,155,574 people (560,942 males, 594,609 females, and 23 intersex) [24]. The county has 353 health facilities spread across the seven subcounties; 41.6% (147 of 353) are public health facilities. Kisumu central subcounty has 117 health facilities, making 33% (117 of 353) of the health facilities in Kisumu County and 0.82% of the total number in the country.

**Figure 2:**
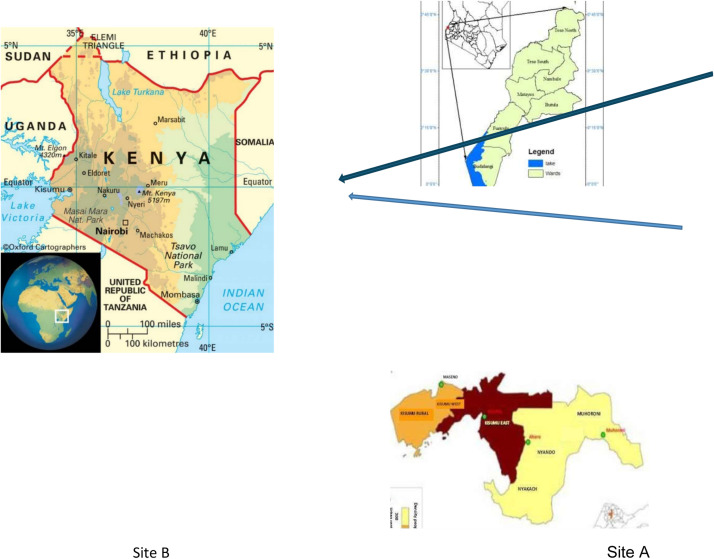
Map of Kenya showing study sites: (Kisumu [A], Busia [B]) site A and site B.

Busia County (site B) is located east of the border town of Busia, Uganda and borders Lake Victoria to the southwest, Siaya County to the southeast, and Bungoma and Kakamega Counties to the east. The county is composed of six subcounties and has a population of 893,681, 426,252 of whom are female and 467,401 are male, according to the 2019 census. The climatic conditions of this county are greatly affected by Lake Victoria. It is inhabited by the Iteso and the Luhya cultural communities (made up of different subtribes). The county has a total of 81 health facilities, and the common diseases include malaria, respiratory diseases, and diarrhea (Figure 2).

### Study design

This was a prospective cohort study among children aged below 5 years presenting with acute febrile illnesses.

### Study population

The study populations included children aged less than 5 years seeking treatment at the level 2 (dispensaries), level 3 (health centers), and level 4 (county hospitals) at the outpatient department in public health facilities at the Busia and Kisumu Counties study sites.

### Sampling and sampling procedures

Bunyala and Kisumu central subcounties in Busia and Kisumu County, respectively, were purposively selected. Bunyala subcounty experiences frequent flooding, whereas while Kisumu central subcounty is the most urbanized and harbor most of the informal settlements, which are normally affected by the flooding and acute water shortages during severe droughts. Flooding and drought are events of climate change, which encourages multiplication of the mosquito, the vector transmitting the diseases. All the level 2, 3, and 4 public health facilities in these regions were also purposively selected. All the children presenting with acute febrile illness in these facilities were all screened. A simple random sampling technique was used to pick the participants among those who screened positive for the diseases.

### Sample size

A total of 1004 febrile children were screened to obtain the desired sample size of 380 and recruited into the study.

### Data collection

All the research assistants were trained on the tools and diagnostic procedures. The data collection tools were also pre-tested in a different health facility before actual data collection to ensure validity and reliability. The study was explained to the parents/guardians at the outpatient department by the study nurse at the triage. If they were willing to participate, then they were sent to the laboratory for screening. If the participant was qualified to be included in the study, the research assistant administered the questionnaire to the parent/guardian, including capturing the demographic characteristic of the participants.

### Laboratory procedures

The malaria and dengue testing were done by trained laboratory personnel working at the selected health facility. The study staffs were trained on both rapid diagnostic tests (RDTs) before the start of the study. A finger stick technique was used to obtain whole blood from the participant’s finger. Testing was done as described in the subsequent testing platforms.

### Dengue fever screening

The OnSite (CTK Biotech, Inc., CA, USA) Duo Dengue antigen (Ag)-immunoglobulin (Ig)G/IgM RDT was used for dengue fever screening. This is a lateral flow immunoassay for the simultaneous detection and differentiation of IgG anti-dengue virus, IgM anti-dengue virus, and dengue NS1 antigen (DENV-1, -2, -3, and -4) in whole blood. The test strip consists of (i) a colored conjugate pad containing recombinant dengue envelope antigens conjugated with colloidal gold (dengue Ag conjugates) and a control antibody conjugated with colloidal gold and (ii) a nitrocellulose membrane strip containing two test lines (G and M lines) and a control line (C line). The G line is pre-coated with antibodies for the detection of anti-DENV IgG, the M line is pre-coated with antibodies for the detection of anti-DENV IgM, and the C line is pre-coated with a control line antibody. The validity of the test was checked by the appearance of a control line on each strip. The Ag test has sensitivity of 100% (95% confidence interval [CI]: 96.8-100%) and a specificity of 99.6% (95% CI: 97.6-99.9%). The IgG test has sensitivity of 97.3% (95% CI: 86.2-99.5%) and a specificity of 99.3% (95% CI: 97.5-99.8%), whereas the IgM test component has a sensitivity of 96.9% (95% CI: 84.3-99.4%) and a specificity of 98.9% (95% CI: 96.9-99.6%) [25].

### Malaria screening

A CareStart Malaria Pf (histidine-rich protein 2) Ag RDT (Access Bio, Inc. NJ, USA) (multi-kit with capped lancet and inverted cup specimen transfer device), a Ministry of Health–approved Malaria Rapid Diagnostic Test, was used to perform the malaria testing at the facility. The kit is a lateral flow immuno-chromatographic antigen-detection RDT, which relies on the capture of dye-labeled antibodies to produce a visible band on a strip of nitro-cellulose, often encased in plastic housing, referred to as cassettes. With malaria RDTs, the dye-labeled antibody first binds to a parasite antigen, and the resultant complex is captured on the strip by a band of bound antibody, forming a visible line (T line) in the results window. One drop of whole blood sample was added to the designated sample well on the cassette. Three drops of assay buffer were added to the developer well. The test was timed for 20 minutes before results were documented. A control line (C line) gave information on the integrity of the antibody-dye conjugate. This test uses a pair of antibodies to detect *P. falciparum* HRP2 only and hence not able to detect non-*P. falciparum* (*P. vivax, P. ovale, and P. malariae*) infections because they do not detect plasmodium lactate dehydrogenase. This test has sensitivity ranging from 88.55% to 100% and specificity from 96.15% to 100%, depending on the study and the parasite density [26].

### Data management and analysis

Data were automatically transmitted and stored in a study password-protected computer. Data cleaning was done, and the data were analyzed using StataCorp 15. The chi-square and Fisher exact test was used to compare categorical variables. Descriptive statistics included sociodemographic characteristics of the participants. Inferential analysis, such as logistic regression analysis, was used to test the significant association between variables at *P* ≤0.05. Odds ratios (ORs) were calculated with 95% CIs at a *P* = 0.05. Statistical data on the prevalence of dengue, malaria, and dengue–malaria coinfection was used for analysis of the correlation between prevalence and the study sites.

### Ethical approval

All the study procedures performed in this research were done in accordance with the ethical standards and the National Commission for Science in Kenya and the 1964 Helsinki Declaration and its later amendments or comparable ethical standards. The research was approved by the Institutional Scientific Ethics Review Committee of Jaramogi Oginga Odinga Teaching and Referral Hospital ref no. ISEC/JOOTRH/752/23) and licensed by the National Commission for Science, Technology, and Innovation (NACOSTI), license No. NACOSTI/P/23/32018. Authorizations were also granted by the county governments of Kisumu (ref: GN133VOL.XV/250) and Busia (ref: CG/BSA/H/ADM/1/56/IX).

## Results

The overall disease burden (malaria and dengue fever) is 37.8% and the prevalence of *P. falciparum* alone infection was 21.4%, dengue alone infection was 8.9%, and coinfections was 7.5% (Table 1).

**Table 1:** Distribution of *Plasmodium falciparum* and dengue fever and co-infections prevalence across counties.

County	Total screened	Pf	DV	Mixed infection
Busia	505	118(23.4%)	27(5.3%)	45(5.3%)
Kisumu	499	97(19.4%)	63(12.6%)	30(9.0%)
**Overall**	**1004**	**215(21.4%)**	**90(8.9%)**	**75(7.5%)**

**Figure 3:**
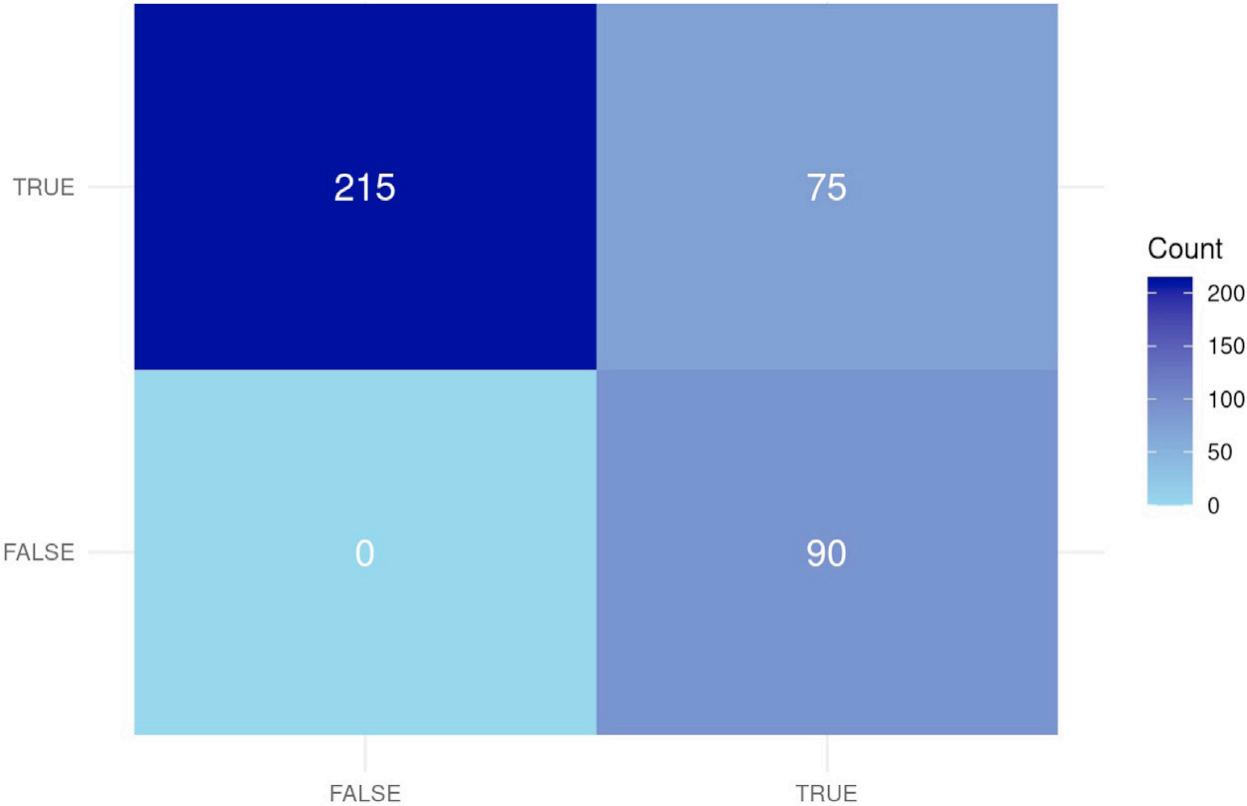
Confusion matrix heatmap illustrating binary co-occurrence of dengue and malaria diagnoses among children under 5 years in Busia and Kisumu Counties.

Of the 380 children, 90 were diagnosed with dengue fever alone (dengue-positive, malaria-negative), 75 with dengue and malaria (coinfection), and 215 with malaria alone (malaria-positive, dengue-negative) (Figure 3).

**Figure 4:**
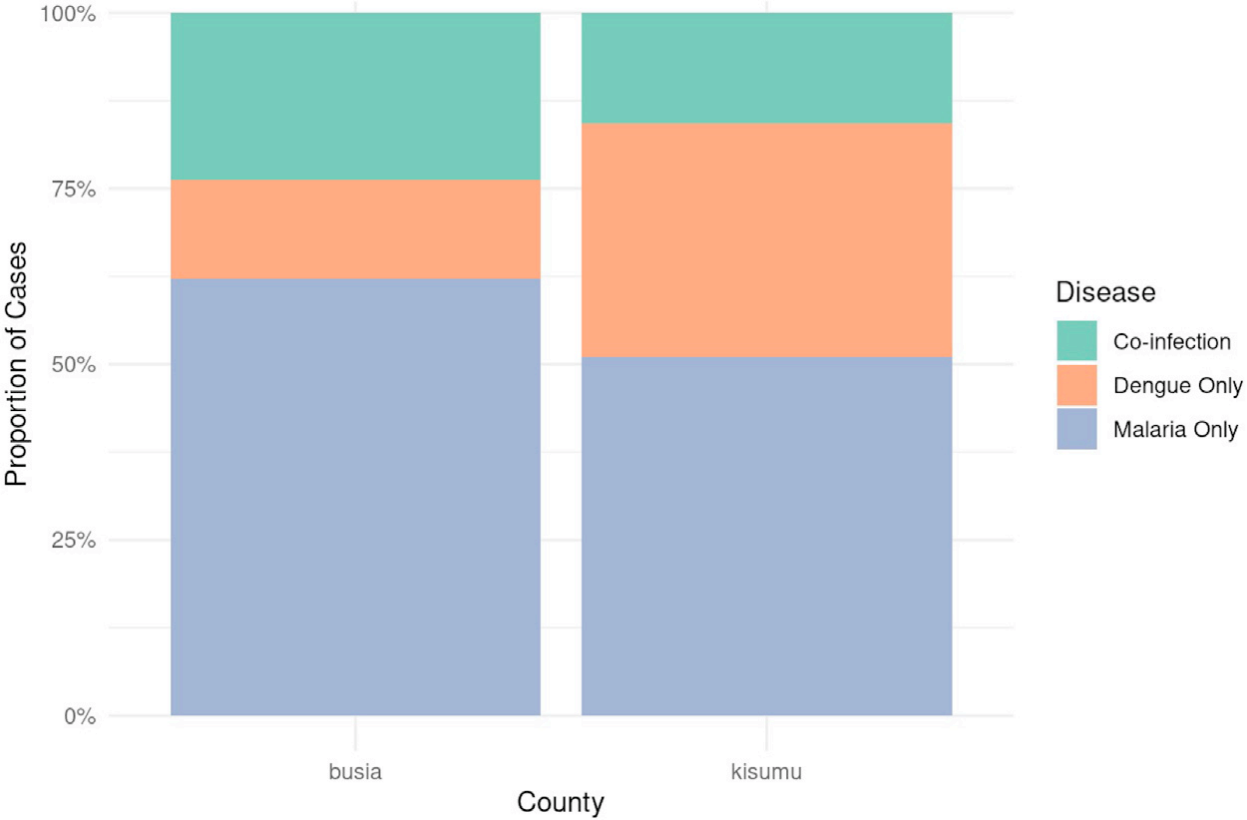
Proportions of dengue and malaria diagnoses within each county (study site).

Busia County, 14.2% (27 of 190) of children aged under 5 years were diagnosed with dengue fever alone, 23.7% (45 of 190) with coinfections, and 62.1% (118 of 190) with *P. falciparum* alone. In contrast, Kisumu County shows a higher proportion of dengue-only cases of 33.2% (63 of 190), a lower coinfection rate of 15.8% (30 of 190), and *P. falciparum* alone infection of 51.1% (97 of 190). There was a relatively higher burden of dengue fever in Kisumu, whereas Busia experiences more *P. falciparum* alone and coinfection cases (Figure 4).

A chi-square test of independence between counties and diagnoses yielded a statistically significant result (X² = 19.45, *P* <0.001), indicating that the distribution of these diagnoses is not random across the counties but rather associated with the location. In addition, the correlation between having malaria and having dengue was negative (r = −0.636), implying that the two serostatuses tend not to occur independently; when one is present, the other is less likely, at least in the overall trend.

Further analysis of coinfection patterns within each county confirmed this tendency. In Busia, 45 participants had malaria and dengue, whereas in Kisumu, 30 participants were coinfected. Interestingly, no participant was diagnosed with dengue fever alone, indicating a strong overlap between the presence of dengue and malaria in this data set or possibly the absence of dengue-only diagnostics or reporting. This is also supported by the OR of zero for having dengue without malaria, further suggesting the rarity or absence of isolated dengue cases in this population.

When looking at the prevalence, 37.9% of participants in Busia had dengue (all in combination with malaria), compared with 48.9% in Kisumu. Malaria prevalence, however, was significantly higher in Busia at 85.8% than in Kisumu at 66.8%. The chi-square tests for county level showed statistically significant differences for dengue (*P* = 0.038) and malaria (*P* < 0.001), reinforcing the observation that disease patterns vary meaningfully between the two locations. Regarding coinfection with both diseases, the overall prevalence was 19.7%. When broken down by county, 23.7% of participants in Busia were coinfected, compared with 15.8% in Kisumu. These results suggest that although dengue is more prevalent in Kisumu, the cooccurrence of both diseases is more common in Busia, pointing to possible differences in environmental or vector control factors, health system diagnostics, or population vulnerabilities.

## Discussion

The goals of this study were to describe the correlation and epidemiologic distribution of the emerging coinfections of *P. falciparum* and dengue fever among febrile children seeking health care in western Kenya. The study showed that the overall seroprevalence of malaria and dengue fever of among the children aged below 5 years was 37.8% (380 of 1004), dengue fever infections alone was 8.9%, *P. falciparum* infections alone was 21.4%, and coinfections was 7.5%. This coincides with a study by Khan *et al.* [27] among children in Kenya, which also demonstrated a significant overlapping of DENV, chikungunya virus, and malaria, especially in densely populated centers, with household crowding and surrounding litter as risk factors for transmission. Others studies have also concluded that dengue and malaria are endemic in all continents [28]. A study by Vohra *et al.* [1] associated the rising cases of dengue and malaria in areas of Pakistan with flooding. This study agrees with this finding because it was done in flood prone areas of western Kenya. Floods affect reproduction, growth, behavior patterns, and population dynamics of arthropod vectors such as mosquitoes [1]. This study found a higher prevalence of malaria compared with dengue, which contradicts the finding of a study by Rao *et al.* [16], which reported higher rates of dengue infection than malaria infection, even though they also reported dengue–malaria coinfection as common in such localities. Dengue and malaria coinfections have been reported in other studies.

This study reveals a relatively higher burden of dengue fever in Kisumu, which is a more urban setting, than Busia, which experiences more concurrent infection. This finding agrees with other studies which have alluded to the proliferation of *Aedes aegypti* in urban environments mediated by the availability of key aquatic habitats. The presence of dengue in both sites confirms that *Aedes aegypti* is well-adapted to and successfully distributed in artificial and natural habitats present in urban environments [29].

This study used RDT for screening, which might not be sensitive enough to enable the detection of all dengue infections; however, the large gap between the proportion of dengue mono-infection and malaria–dengue coinfection in a random sample of febrile patients could still be informative. Although enzyme-linked immunosorbent assay is recommended for the confirmation of dengue suspected cases, RDTs are still useful tools for dengue screening in limited resource settings with limited or unavailable reference diagnostic services [7]. Similarly, this study being hospital-based is limited by the fact that it recruited febrile children. Therefore, the findings may not be generalizable and do not reflect the epidemiologic status at the community level. However, the findings can be used as a basis for further large-scale community-based studies in coendemic areas for both types of infection.

The fact that seropositive DENV cases could be detected suggests that endemic transmission is actively ongoing in Kenya [30]. The findings of this study have significant public health policy implications. The observed concurrent serostatus further worsen the concern that, in the absence of routine testing for DENV, malaria-attributed morbidity and mortality may be due to associated misdiagnosed coinfections. Such misclassification can have impact on public health decision-making. According to a study by Vu *et al.* [31], the detection of acute DENV infection highlighted the inequitable access to diagnostic testing, which is a fundamental flaw in global surveillance for DENV in resource-restricted settings and inaccurate epidemiologic data; this misdirects the use of limited resources for public health interventions. In this study, the high prevalence of dengue, malaria, and dengue–malaria coinfection suggested that the region could be becoming endemic for both diseases, which vary according to the coexistence of the vectors, the environment, and climatic factors, including the optimum temperature, with a high relative humidity and abundance of fresh water lakes.

## Conclusion

Dengue and malaria coinfection is an arising mosquito-borne infection that causes febrile illnesses among children. The coinfection is an indication of a potential overlap and challenges in diagnosis and management. The highest temperature, precipitation, and humidity experienced in the region is favoring vector fecundity, calling for a strengthened surveillance and formulation of strategies, with emphasis on vector control methods to reduce the transmission and future epidemics. It is also important to educate clinicians on differential diagnoses between dengue viral infection and malaria infection for appropriate case management because the serostatuses for these two infections may not be clinically distinguishable. Future, multi-country, prospective studies are needed to understand the effect and spread of coinfection on the severity of the disease and transmission at a global community level.

## Declaration of competing interest

The authors have no competing interests to declare.
